# Acute and Chronic Effects of Interval Training on the Immune System: A Systematic Review with Meta-Analysis

**DOI:** 10.3390/biology10090868

**Published:** 2021-09-03

**Authors:** Daniel Souza, Arthur F. Vale, Anderson Silva, Murilo A. S. Araújo, Célio A. de Paula Júnior, Claudio A. B. de Lira, Rodrigo Ramirez-Campillo, Wagner Martins, Paulo Gentil

**Affiliations:** 1 Faculdade de Educação Física e Dança, Universidade Federal de Goiás, Goiânia 74690-900, Brazil; daniel_souza86@discente.ufg.br (D.S.); arthur_vale@discente.ufg.br (A.F.V.); andersongarcia@discente.ufg.br (A.S.); muriloaugusto@discente.ufg.br (M.A.S.A.); andre.claudio@gmail.com (C.A.B.d.L.); 2 Centro Universitário Araguaia, Goiânia 74223-060, Brazil; celiopersona@gmail.com; 3 Department of Physical Activity Sciences, Universidad de Los Lagos, Santiago 8320000, Chile; r.ramirez@ulagos.cl; 4 Faculdade de Fisioterapia, Universidade de Brasília, Distrito Federal, Brasilia 70910-900, Brazil; professorwagnermartins@gmail.com

**Keywords:** immunity, immunologic monitoring, immunoglobulin A, aerobic capacity, physical activity, high-intensity interval exercise, leukocytes, infectious disease

## Abstract

**Simple Summary:**

Interval training (IT) is a popular training strategy recognized by its positive effects on metabolic and cardiovascular system. However, there seems no consensus regarding the effects of IT on immune system parameters. Therefore, we aimed to summarize the evidence regarding the effects of IT on the immune system. As our many findings, an IT acutely promote a transitory change on immune cell count followed by reduced function. The magnitude of these changes seems to vary in accordance with IT type. On the other hand, the regular practice of IT might contribute to improve immune function without apparent change on immune cell count.

**Abstract:**

Purpose: To summarize the evidence regarding the acute and chronic effects of interval training (IT) in the immune system through a systematic review with meta-analysis. Design: Systematic review with meta-analysis. Data source: English, Portuguese and Spanish languages search of the electronic databases Pubmed/Medline, Scopus, and SciELO. Eligibility criteria: Studies such as clinical trials, randomized cross-over trials and randomized clinical trials, investigating the acute and chronic effects of IT on the immune outcomes in humans. Results: Of the 175 studies retrieved, 35 were included in the qualitative analysis and 18 in a meta-analysis. Within-group analysis detected significant acute decrease after IT on immunoglobulin A (IgA) secretory rate (*n* = 115; MD = −15.46 µg·min^−1^; 95%CI, −28.3 to 2.66; *p* = 0.02), total leucocyte count increase (*n* = 137; MD = 2.58 × 10^3^ µL^−1^; 95%CI, 1.79 to 3.38; *p* < 0.001), increase in lymphocyte count immediately after exercise (*n* = 125; MD = 1.3 × 10^3^ µL^−1^; 95%CI, 0.86 to 1.75; *p* < 0.001), and decrease during recovery (30 to 180 min post-exercise) (*n* = 125; MD = −0.36 × 10^3^ µL^−1^;−0.57 to −0.15; *p* < 0.001). No effect was detected on absolute IgA (*n* = 127; MD = 47.5 µg·mL^−1^; 95%CI, −10.6 to 105.6; *p* = 0.11). Overall, IT might acutely reduce leucocyte function. Regarding chronic effects IT improved immune function without change leucocyte count. Conclusion: IT might provide a transient disturbance on the immune system, followed by reduced immune function. However, regular IT performance induces favorable adaptations on immune function.

## 1. Introduction

It is widely accepted that moderate-intensity continuous training (MICT) with short to moderate duration (<60 min) is associated with an enhanced immune defense [1]. However, acute bouts of high-intense or high-volume aerobic exercise might provide transitory negative changes on immune cell count and function (lasting between 3 h to 72 h depending on the immune outcome) [2,3]. This might lead to immunosuppression and increased risk for infectious diseases [1,4,5].

The underlying mechanisms to exercise-induced immunosuppression, referred to as the “open window”, are multifactorial and involve neuroendocrine and metabolic factors such as catecholamines, cortisol and growth hormones [3,6]. Immunosuppression usually occurs after intensive training protocols that result in increased levels of inflammation, metabolic and oxidative stress [4]. Therefore, it is important to study different aerobic training protocols since different physiological demands could have different impacts on immune function.

Interval training (IT) is an aerobic training strategy that usually consists in interspacing periods of high-intensity efforts with periods of rest or low-intensity exercise [7,8]. The rationality behind this strategy is to allow the accumulation of higher volume of vigorous exercise than those that could be achieved performing continuous exercise at high intensity [9]. Although current studies about the topic involve low-volume protocols [10,11,12,13], IT is usually performed near or at maximum individual’s capacity, which might result in higher metabolic and hormonal stress in comparison with MICT [14].

During the past century, IT gained popularity in sports preparation [15]. This training strategy was widely adopted by coaches and athletes to train at workloads closer to their specific performance competition [15]. However, in recent decades the recommendations of IT performance have been extended to non-athlete’s subjects as an effective strategy for health promotion [16,17]. Although compelling evidence from healthy and clinical populations have consistently shown that IT promotes metabolic and cardiovascular benefits in a similar or greater extent than MICT [10,18,19,20,21], there seems to be no consensus regarding the effects of IT on the immunological system.

Previous studies suggest that IT might induce changes in immune function for a few hours after exercise cessation [22,23,24,25,26]. There is evidence of both positive [27,28] and negative [29,30] functional adaptation of the immune system in response to repeated IT sessions such as improvements on immune defense and reduced immune cell count or death. Considering these controversial findings, it remains to be elucidated how IT strategies might affect the immune system, especially considering the many different IT models [7]. This knowledge might contribute to optimize IT prescription in both health and disease, elucidating which aspects in IT prescription might impact on immune system modulation and help health professional to prescribe more efficient and safer exercise protocols to different populations. Therefore, we aimed to summarize the evidence through a systematic review of literature regarding the results of clinical trials that investigated the acute and chronic effects of IT on immune measures in humans. Additionally, a meta-analysis was conducted to determine the acute effects of IT on the relevant immune parameters that presented sufficient data.

## 2. Methods

The set of items of this systematic review are reported according to the Preferred Reporting Items for Systematic Reviews and Meta-Analyses (PRISMA) statement [31]. The study protocol was registered with the International Prospective Register of Systematic Review (PROSPERO; available at: https://www.crd.york.ac.uk/PROSPERO/, accessed on 5 July 2020) (registration number CRD42020176291) [32]. The study design followed PICO strategy: humans (Population), acute or chronic IT (Intervention), other exercise interventions, non-exercise control or without comparison group (Comparison), leucocyte count, neutrophil count or function, lymphocyte count or function (Outcomes).

### 2.1. Eligibility Criteria

Systematic search comprised studies such as clinical trials, randomized clinical trials and randomized cross-over trials in humans. Studies were considered eligible for inclusion according to the following criteria: (i) involved humans without restriction for age, sex or health condition (ii) involved at least one IT session (with comparison group or not), here defined as intermittent activities that interspersed maximal efforts (e.g., all-out sprints) or close to maximal efforts (≥80% of peak or maximal oxygen uptake ($\dot{V}$O_2peak/max_) or ≥85% of peak or maximal heart rate (HR_peak/max_)) with passive or activity recovery [33]; (iii) appropriate measures of exercise intensity (i.e., heart hate, $\dot{V}$O_2_, performance markers, or rating of perceived exertion); (iv) investigated at least one outcome of acute or chronic IT intervention on innate or acquired immune system; (v) published in English, Spanish or Portuguese. Studies were excluded based on the following criteria: (i) clinical trial registers or non-concluded studies, dissertation and thesis, letter to editor, literature reviews and observational studies; (ii) involved concurrent (i.e., IT combined with resistance training) or polarized training (i.e., IT combined with MICT).

### 2.2. Search Strategy

English, Portuguese and Spanish language searches of the electronic databases Pubmed/Medline, Scopus, SciELO were initially performed in March 2020 with an update on November 2020. Articles were retrieved from electronic databases using key words and MeSH terms: (“high intensity interval training”) OR “high intensity intermittent training”) OR “high intensity interval exercise”) OR “high intensity intermittent exercise”) OR “sprint interval training”) OR “repeated sprint training”) OR “hiit”) OR “interval training”)) AND (“immune system”) OR “neutrophil”) OR “iga”) OR “immunoglobulin”) OR “macrophage”) OR “monocyte”) OR “leucocyte”) OR “lymphocyte”) OR “upper respiratory tract infection”) OR “urti”) OR “illness”) OR “immunity”). To inception, retrieved articles on systematic search were checked for relevance by two independent researchers (DS and AFV). After excluding repeated references, articles were selected after a sequenced title and abstract reading, always in this order. The agreement rate between reviewers for the title/abstract screening was high (kappa = 0.944, *p* < 0.001). After, DS and AFV independently reviewed the full texts of potentially eligible papers, and a third researcher (PG) was consulted when there was any disagreement between reviews. Additionally, manual search was conducted through to the references of all included studies to obtain an integrative cross-references full-text selection.

### 2.3. Data Extraction

The following data were independently extracted by two authors (DS and AFV): study design, participants characteristics (age, sex, sample size, health status, level of physical conditioning), IT protocols description and outcomes of innate and acquired immune measures (leucocytes, neutrophils, lymphocytes, lymphocyte subsets, monocytes, eosinophil, basophil, granulocyte, immunoglobulin A (IgA)). Additionally, IT was classified in accordance with training characteristics. The IT protocols involving maximal sprints (“all out” effort) were classified as sprint interval training (SIT), while IT protocols involving submaximal efforts such as the intensities closer to those that elicit the maximum oxygen consumption (≥85% $\dot{V}$O_2max_) were classified as high-intensity interval training (HIIT). Studies were classified as acute and chronic interventions in accordance with their respective characteristics. Acute studies were defined as those that investigated the acute effects (usually transitory lasting up to 48h after exercise cessation) provided by a single IT session. While the chronic studies were defined as those that investigated the adaptations provided by accumulated IT sessions (at minimum 3 sessions). Parameters such as immune cell count referred to the quantity of immune cell, while immune cell function is associated with the immune response against stressor agents.

### 2.4. Study Quality

Study quality of randomized controlled trials that met inclusion criteria was independently assessed by two authors (AS and AFV) using the Tool for the Assessment of Study Quality and Reporting in Exercise (TESTEX) scale. The TESTEX scale is a validated tool (ICC ≥ 0.91, *p* < 0.001) specifically constructed for assessing the methodological quality of studies on physical exercise and training. This scale is composed by 15 points (5 points for studies quality and 10 points for methodologic reports) [34].

The scale considers this criteria’s for punctuation: eligibility criteria specified (1point); randomization specified (1 point); allocation concealment (1 point); groups similar at baseline (1 point); blinding of assessor (1 point); measure at last one primary outcome in 85% subjects (until 3 points); intention to treat analyses (1 point); compare groups in at last one primary outcome (until 2 points); all outcomes are reported with points estimates (1 point); control patients are asked to report their levels of physical activity and their data are reported (1 point); exercise load is titrated to keep relative intensity constant (1 point); exercise volume and energy expenditure can be calculated (1 point) [34]. Thus, a 15-point maximum score can be obtained by each study. Additionally, we adopted a study quality classification adapted from previous systematic review with meta-analysis [21], where the punctuation obtained from each study was divided by 15 and subsequently multiplied by 100, resulting in a study quality expressed as percentage. Based on this, study quality was classified as low (<50%), fair (between 50% and 66.6%), and high (>66.6%). The study quality based on TESTEX punctuation was not used as exclusion criteria.

### 2.5. Statistical Analyses

A meta-analysis was conducted to determine the overall acute effects of IT on the immune outcome that presented a minimum of five trials, such as IgA concentration (µg·mL^−1^), IgA secretory rate (µg·min^−1^), total leucocyte and lymphocyte count (10^3^ µL^−1^). The number of results regarding the chronic effects of IT on a specific immune outcome was not sufficient to perform a meta-analysis. The effects for meta-analysis were calculated using the pre-intervention to post-intervention mean change and were presented as mean difference (MD) and 95% confidence interval. When the pre- and post-intervention values were not reported, the study was excluded from the meta-analysis. If the values were available only in figure, the authors were contacted by email to data request. When the response was not provided, the numeric data was obtained from chart through data extraction software (GraphData 1.0, Brazil). To evaluate the biphasic characteristic of lymphocyte count (i.e., immediately increase followed by decrease), an additional effect was calculated pre-exercise to the first time point recovery immediately post-exercise (i.e., obtained between 30 and 180 min post-exercise). Random-effects model was preferred due the high methodological variation between studies. The meta-analysis between conditions (IT vs. non-exercise) was not performed due too few studies involved a non-exercise arm as control condition. Statistical heterogeneity of the treatment effect among studies was tested using the Chi-square test and the inconsistency I^2^ test, in which *p* < 0.10 and values above 50% were considered indicative of substantial heterogeneity. A sensitivity analysis was conducted to determine the contribution of each study to the overall effect by successively removing de results of each study and using the data from the remaining studies. In addition, subgroup analyses were performed to detect the influence of participants sex, modality, or IT type. Analyses were conducted using the Review Manager software (RevMan 5.3, Nordic Cochrane, Denmark), and the accepted level of significance was (*p* < 0.05).

## 3. Results

### 3.1. Included Studies

**Figure 1:**
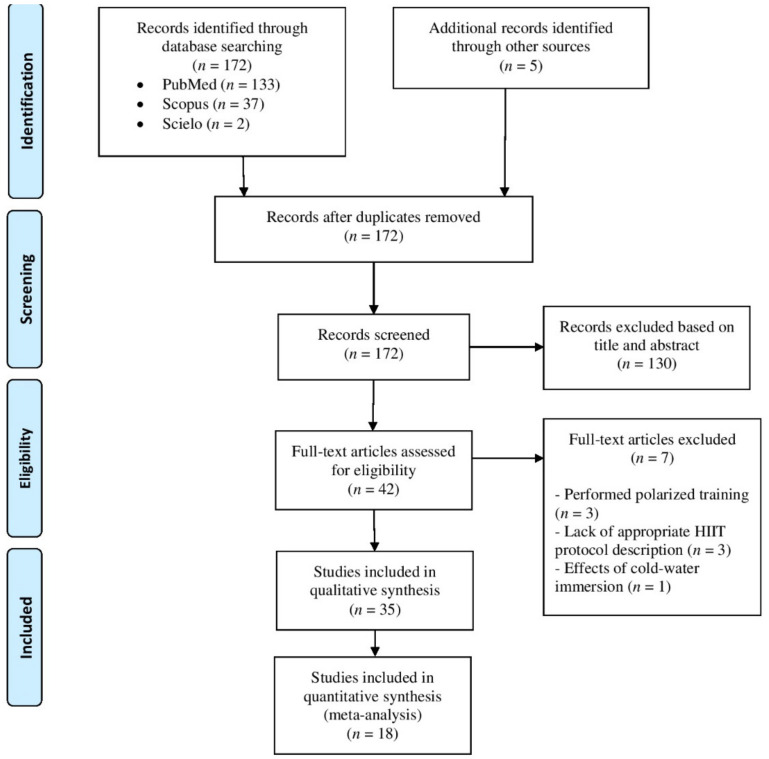
Flowchart of study selection.

Initially, 174 records were retrieved through searches strategy. After removing duplicates, 172 articles were screened for title and/or abstract analyses. Within these, 130 studies did not meet inclusion criteria and were removed. Subsequently, two researchers (DS and AFV) independently reviewed full text of the 42 remaining studies, in which three studies were removed because involved polarized training [28,35,36], three studies lack appropriate IT protocol description [37,38,39] and one study involved cold water immersion [40]. As result, 35 studies were included in final qualitative analysis. From these, 18 studies were included in quantitative analysis, where 12 studies were clinical trials, and six studies were randomized cross-over trials. When the study involved more than one IT intervention (e.g., different IT protocol or separated by sex), the data obtained from each intervention was calculated as an independent trial in meta-analysis. All these steps are described in Figure 1.

### 3.2. Summary of Studies

Studies’ characteristics are summarized in Table 1 and Table 2. Twenty-three studies investigated exclusively the acute effects of IT, whereas 10 studies performed interventions lasting between 1 [29,41] and 26 weeks [42]. Two studies performed both acute and chronic investigations [23,27].

Among the 35 included studies, the numbers of participants by IT interventions ranged from 7 [41] to 50 [52], for a total of 509 participants from both sexes. Twenty-four studies involved exclusively men, three studies involved exclusively women [22,54,63] and eight studies investigated mixed-sex samples [29,42,43,52,53,62,64,66]. Participants’ age varied from 15.5 ± 0.6 [54] to 64.0 ± 7.0 years [64]. In most studies participants were apparently healthy, with the exception of studies that involved overweight-obese men [41,46,51], elderly with rheumatoid arthritis [64] and elderly with prediabetes [62]. The training status of the participants varied from sedentary with clinical conditions to high-performance athletes.

### 3.3. Intervention Characteristics

Regarding training intervention, the included studies used a diversity of modalities and IT protocols. Most used cycling (*n* = 18) or running (*n* = 11), while some studies used walking [62,64], arm-cycling [52], paddling [60,65] and swimming [58]. Twenty-five studies involved exclusively submaximal IT protocol (i.e., HIIT), 10 studies involved exclusively maximal IT protocol (i.e., SIT), usually Wingate-based protocols (i.e., repeated 30 s “all-out” effort) and one study involved both submaximal and maximal protocols [44], and compared different rest interval mode (e.g., passive or active) for HIIT and SIT.

The intensity of HIIT protocols was prescribed and controlled based on percentage of maximal velocity achieved during incremental test (Vmax) [29,30,46,60,61], velocity associated with $\dot{V}$O_2max_ [43,59] or $\dot{V}$O_2peak_ [65], percentage of $\dot{V}$O_2max_ [50,55,56,57,67], or reserve oxygen uptake ($\dot{V}$O_2reserve_) [62,64], percentage of HR_max_ [41,66] or reserve heart rate HR_reserve_ [42], and percentage of peak power [25,26,44,51,58] or maximum anaerobic power [27]. Eleven studies prescribed SIT protocol using “all out” efforts [22,23,24,44,45,47,48,52,53,54,63]. Characteristics of IT protocols are detailed in Table 1 and Table 2.

### 3.4. Qualitative Analysis of Acute Effects of IT on Immune Outcomes

#### 3.4.1. Salivary Immunoglobulin A

**Figure 2:**
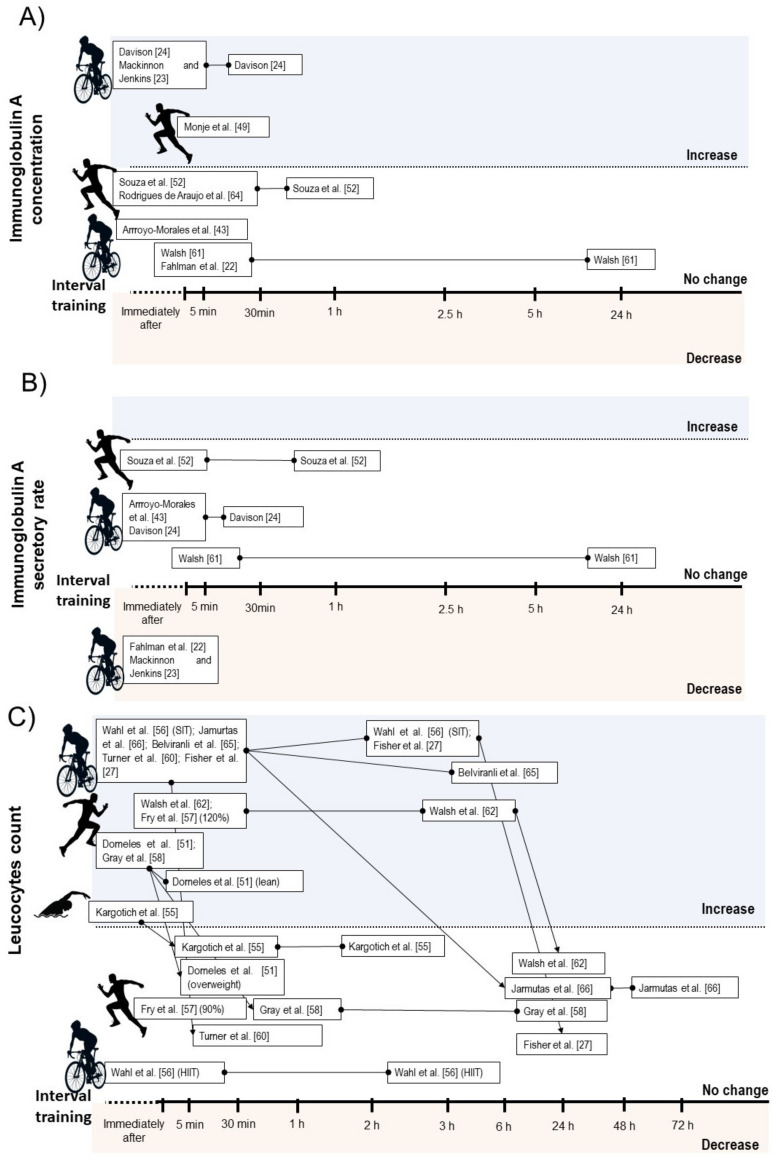
Illustration of time course of salivary immunoglobulin A concentration (**A**), salivary immunoglobulin secretory rate (**B**), and total leucocyte count (**C**) after acute interval training session.

A qualitative description of the acute effects of IT on immune measures are presented in Table 1. Six studies verified no change on absolute salivary IgA concentration after IT [22,46,47,52,54,55], while three studies verified transitory increase lasting up to 30 min after exercise [23,24,43]. Regarding secretory rate of IgA, four studies verified no change [24,46,52,55], and two studies verified decrease after exercise [22,23]. Considering IT type, the acute decrease on IgA secretion rate was only observed after SIT [22,23], while no HIIT intervention reduced this parameter [46,52,55] (Figure 2B).

#### 3.4.2. Leucocyte Count

Ten studies verified transitory increases in total leucocyte count lasting up to 6 h after SIT [44,45,48] or HIIT [27,50,51,56,58,59,61]. One study verified no change on leucocyte count after a HIIT protocol with passive or active recovery [44]. Additionally, Fry et al. [61] reported a significant increase on leucocyte count immediately after HIIT when the high-intensity bouts were performed at 120% of V_max_, but not at 90% (Figure 2C).

**Figure 3:**
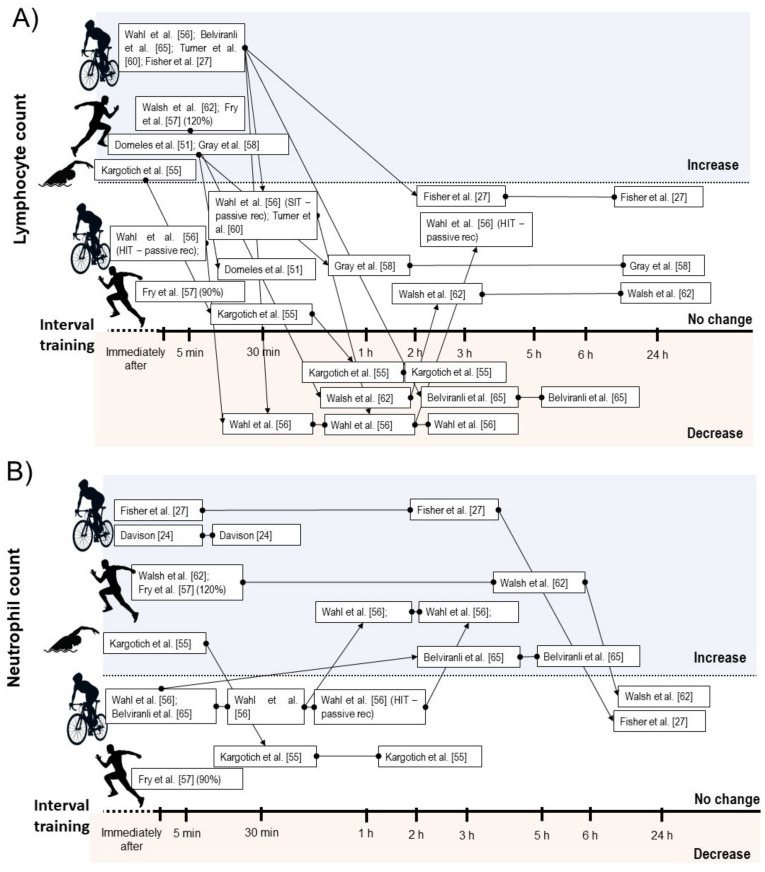
Illustration of time course of lymphocyte (**A**), and neutrophil count (**B**) after acute interval training session.

Considering leucocyte subsets, nine studies showed increases on total lymphocyte counts immediately after a SIT [48,53] or HIIT session [27,50,51,56,58,59,61], while two HIIT intervention did not change lymphocyte count immediately after exercise [44,61]. Five intervention verified decrease on lymphocyte count between 30 min and 6 h after exercise [44,48,56,58]. From these, two involved SIT [44,48] and three involved HIIT [44,56,58]. Within the studies that did not observed lymphopenia during IT recovery, all involved HIIT [27,50,51,59] (Figure 3A).

Seven studies reported increases on neutrophil count after SIT [24,44,48] or HIIT performance [27,56,58,61]. In some studies, the increased neutrophil count occurred immediately after exercise and remained elevated between 30 min and 5 h [24,27,56], while two studies verified delayed increase in this parameter starting between 1 h and 3 h after exercise [44,48] (Figure 3B). Five studies found increases on monocyte count immediately after SIT [44] or HIIT [51,58,59,61], while two studies involving SIT [48] and HIIT [44] verified no change on this measure. Regarding mixed cell count, two studies reported acute increases on eosinophil and basophil [44,48], and one study verified increases on granulocyte count after HIIT exercise [59]. One study involving HIIT verified no change on eosinophils count [58], while the study by Wahl et al. [44] showed no change and decrease on basophils and eosinophils count after HIIT and SIT protocol, respectively.

#### 3.4.3. Leucocyte Function

Five studies involving HIIT [26,27,57,60,61] reported a transitory reduction in lymphocyte function or reduced cell viability after IT performance (lasting up to 3 h) in response to in-vitro stimulation. One study found mobilization of low differentiated T cells and regulatory T cells (Treg) immediately after HIIT, in parallel with apoptosis of high differentiated T cells three hours after exercise [49]. Two studies verified transitory reduced neutrophil function after SIT [24] and HIIT [25] performance (lasting up to 30 min) in response to in-vitro stimulation (Table 1).

### 3.5. Qualitative Analysis of Chronic Effects of IT on Immune Outcomes

A qualitative description of the chronic adaptations on immune measures in response to IT is presented in Table 2. Two studies involving SIT reported no change on salivary IgA (absolute concentration or secretory rate) after training [23,63]. Three studies involving HIIT found no significant change in leucocyte count [30,42,65]. Regarding leucocyte function, one study verified increases on peripheral lymphocyte T helper subsets (i.e., memory regulatory T cell and Treg) [41] after HIIT. Three studies involving HIIT provided significant improvements on neutrophil function [62,64,66] and two studies involving HIIT [27,67] verified improvements on lymphocyte function. In contrast, a study involving three consecutive days of HIIT performed until exhaustion reported a significant increase on lymphocyte migration and apoptosis after the third day of consecutive training session [29].

### 3.6. Quality Assessment

Considering the specificity of the TESTEX scale, only 14 studies were included in this analysis and the results are shown in the Table 3. The studies achieved an average score of 4.6 from a total of 15 points. Point estimate of outcomes and exercise volume were the most reported features in the included studies. Most studies failed to report if there were, or not, adverse events associated with exercise intervention or intention to treat analysis.

### 3.7. Meta-Analysis

**Figure 4:**
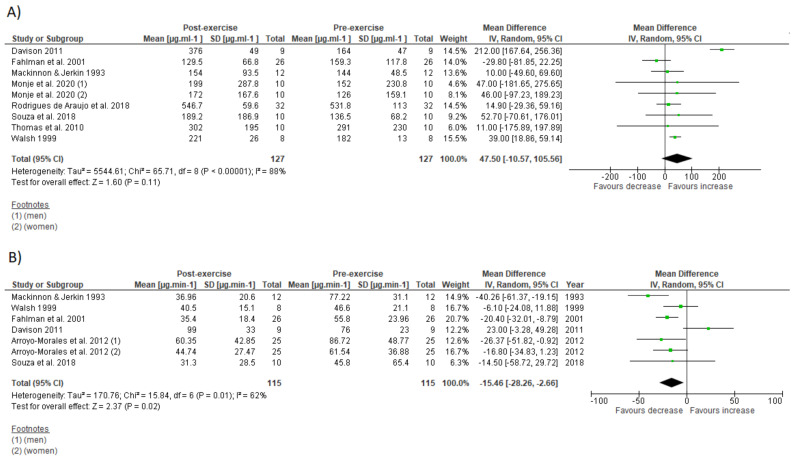
Forest plot of the acute effects of interval training on absolute immunoglobulin A concentration (**A**) and immunoglobulin secretory rate (**B**). SD standard deviation, CI confidence interval, IV random effects. The green squares represent the mean difference for each dataset. The black diamonds represent the estimated overall effect.

**Figure 5:**
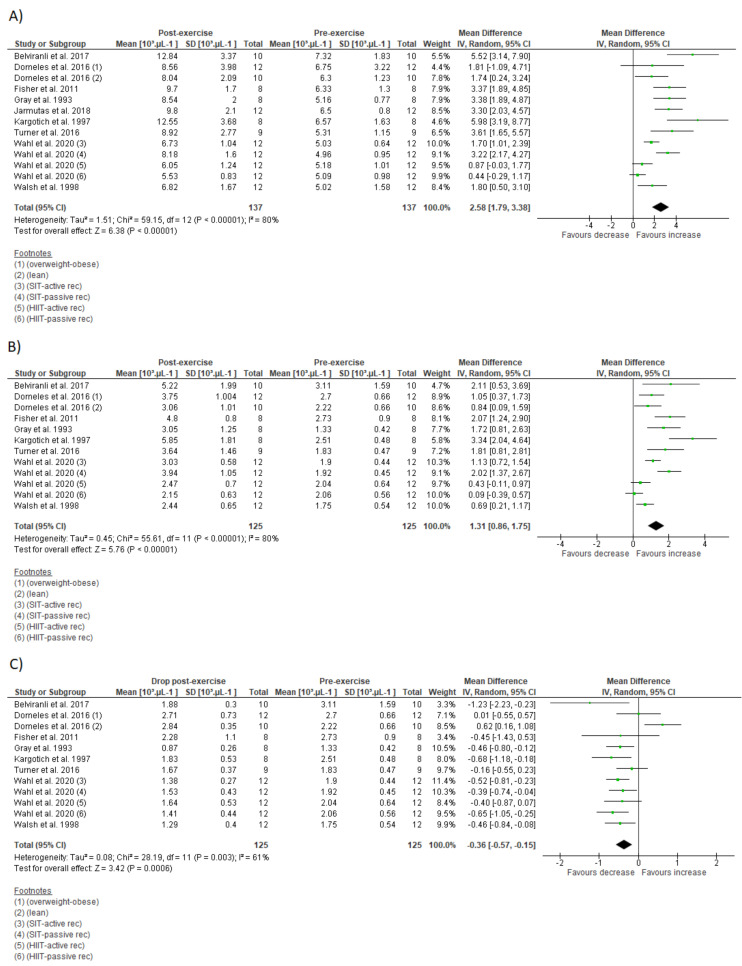
Forest plot of the acute effects of interval training on total leucocytes count (**A**), lymphocyte count immediately after exercise (**B**), and lymphocyte count at first drop during recovery (**C**). SD standard deviation, CI confidence interval, IV random effects. The green squares represent the mean difference for each dataset. The black diamonds represent the estimated overall effect.

The effects of IT on immune parameters are present in Figure 4 and Figure 5. The within-group analysis found that IT significantly reduced IgA secretory rate immediately after exercise (*n* = 115; MD = −15.46 µg·min^−1^; 95%CI, −28.3 to 2.66; ∆% = −24%; *p* = 0.02) (Figure 4B). However, there was no significant change on absolute IgA concentration (*n* = 127; MD = 47.5 µg·mL^−1^; 95%CI, −10.6 to 105.6; ∆% = 23%; *p* = 0.11) (Figure 4A). There was significant increase on total leucocyte count immediately after exercise (*n* = 137; MD = 2.58 × 10^3^ µL^−1^; 95%CI, 1.79 to 3.38; ∆% = 44%; *p* < 0.001) (Figure 5A). Additionally, IT promoted significant increase on lymphocyte count immediately after exercise (*n* = 125; MD = 1.3 × 10^3^ µL^−1^;95%CI, 0.86 to 1.75; ∆% = 60%; *p* < 0.001) (Figure 5B), followed by significant reduction at the first recovery time point after post-exercise (30 to 180 min post-exercise) (*n* = 125; MD = −0.36 × 10^3^ µL^−1^;−0.57 to −0.15; ∆% = −17%; *p* < 0.001) (Figure 5C). Substantial heterogeneity was detected in the analysis for IgA concentration (I^2^ = 88%; *p* < 0.001), IgA secretory rate (I^2^ = 62%; *p* = 0.01), leucocyte count (I^2^ = 80%; *p* < 0.001), lymphocyte count immediately after exercise (I^2^ = 80%; *p* < 0.001), and during recovery (I^2^ = 61%; *p* = 0.003).

Subgroup analysis detected a significant effect of IT type (HIIT vs. SIT) on IgA secretory rate decrease and lymphopenia for SIT, and on absolute IgA concentration increase for HIIT (Table 4). There was a significant effect of participant sex (men vs. women) on IgA secretory rate only for women and training modality (cycling vs. running) on lymphopenia only for cycling.

### 3.8. Sensitivity Analysis

After sensitive analysis performance that checked outlies studies by successively removing the results of each study, changes were observed in effects of IT on absolute IgA concentration (*p*-value ranged from 0.001 to 0.14) and IgA secretory rate (*p*-value ranged <0.001 to 0.1) but not on total leucocyte count (*p* < 0.001), lymphocyte count immediately after exercise (*p* < 0.001), and during recovery (*p*-value ranged from <0.001 to 0.004).

## 4. Discussion

The aim of the present study was to summarize the evidence through systematic review of the experimental studies that investigated the acute and chronic effects of IT on immune measures. The main findings regarding acute studies were: (i) IT compromises IgA secretory rate but not IgA absolute concentrations; (ii) IT promotes transitory leukocytosis (lasting up to 6 h); (iii) IT promotes lymphocytosis followed by transitory lymphopenia (lasting up to 6 h); (iv) IT promotes a transitory impairment on lymphocyte and neutrophil function. Regarding chronic studies: (i) there are no changes on mucosal immune measures (IgA secretory and concentration) after repeated IT sessions spanning from 8 to 12 weeks; (ii) performing IT for 1 to 24 weeks provide no change on leucocyte count; (iii) chronic IT performance promotes favorable adaptations on lymphocytes, monocytes, and neutrophils function.

Both salivary secretory IgA and salivary IgA concentration play a major role in mucosal immune system and their levels have been inversely associated with occurrence of upper respiratory tract infection (URTI) [68,69]. The assumption that a single session of IT could compromise the salivary IgA due its high-intensity nature was supported by the meta-analysis; however, this seems be true only for IgA secretory rate (Figure 2B). Whereas meta-analysis showed significant depression on IgA secretory rate immediately after exercise, there was no change on absolute IgA concentration (Figure 2A). The levels of absolute IgA concentration might even increase after a HIIT session (Table 4).

The substantial heterogeneity observed in the results regarding the acute effect of IT on salivary IgA would be associated with large variety of methodological aspects of studies analyzed. For example, our subgroup meta-analysis revealed a different response of salivary IgA between sex after IT with a significant effect on IgA secretory rate for women but not for men (Table 4). This finding reinforces the role of sex on mucosal immunity modulation [52], which may be associated with differences on hormonal and/or autonomic nervous system activity between sexes [70,71]. Moreover, subgroup analysis detected significant effect on IgA secretory rate for SIT but not for HIIT, which suggest that the different IT types impact differently in this parameter (Table 4). Other methodological issues might also contribute to different results between studies such as dehydration, saliva method collection, or how IgA is expressed, as previously stated [4]. These findings should be interpreted with caution since the sensitive analysis detected the presence of outlier studies.

It is important to note that the acute impairments in salivary secretory IgA rate verified after a single SIT session has not been associated with occurrence of URTI [22,23]. This suggests that the magnitude of the observed transitory depression in salivary secretory IgA after SIT has no clinical relevance. A similar result was also confirmed after 8 weeks of SIT [23], showing that SIT could be performed three times per week in alternated days without altered susceptibility for URTI. Whereas there are some controversial results regarding the acute effects of SIT on salivary IgA secretory rate [22,23,24,52], HIIT seems to not impair IgA secretion rate or absolute IgA concentration in both trained and sedentary populations [43,46,55].

There is evidence that regular practice of IT might confer health and performance enhancing effect (e.g., aerobic and anaerobic capacity) without compromise the mucosal immune system in both athlete [23] and non-athlete population [63] (Table 2). Despite the study by Born et al. [28] involving polarized training and not meeting criteria to be included in this systematic review, it reveals interesting findings regarding the positive adaptation of the mucosal immune function in trained runners after nine HIIT sessions. Considering that the addition of IT into habitual aerobic training routine improved mucosal immune resilience to stress in parallel with the improvements on $\dot{V}$O_2max_ [28], it is reasonable to suggest a relationship between changes in cardiorespiratory fitness and modulation of immune mucosal function.

The leukocytosis observed after a single IT session was supported by our quantitative analysis (Figure 3A). The early and rapid increase in blood leucocyte count after IT session might result from lymphocytosis, as well as detachments of neutrophil and monocytes from blood vessels caused by a high shear stress and catecholamines production, while the prolonged late increase seems to be induced by increases on cortisol levels that release neutrophils from bone marrow [3,4]. However, these responses might differ in magnitude, time course or duration depending on IT type [44], as well as body composition and cardiorespiratory fitness of the participants [51] (Figure 2C).

Whereas neutrophil count increases after an IT session, neutrophil function is transiently reduced in response to in-vitro stimulation [24,25] (Table 1). This functional impairment may be partially explained by the increased release of functionally immature neutrophil from bone marrow or by direct mechanisms such as stress hormones and oxidative stress [24]. However, it is not clear if this transitory functional impairment is clinically relevant. Moreover, neutrophil function is completely restored 24 h after exercise [25]. In contrast to acute findings, chronic IT effects might result in beneficial adaptations on neutrophil function such as improved stimulated reactive oxygen species (ROS) production, improved chemotaxis and reduced basal ROS production in both young and aging people [62,64,66] (Table 2). Recently, Bartlett et al. [62] verified improvements on neutrophil function after 10 weeks of a low-volume IT protocol in people with pre-diabetes.

A common concern regarding intensive aerobic training is its effects on cell-mediated immunity [17]. Although this is still a matter of debate [72], reduced lymphocyte count and function are usually associated with immunosuppression and increased risk for illness [1]. The present meta-analysis verified significant decrease in lymphocyte count during IT recovery (lymphopenia) (Figure 5C). On the other hand, subgroup analysis suggests that HIIT might not necessarily promote lymphopenia, despite its high intensity characteristic (Table 4). Even in absence of lymphopenia, lymphocytes may become more vulnerable to stressful agents few hours after an IT session [26,27,57,60] (Table 1). These transitory functional impairments might be partially explained by changes in lymphocyte subset (e.g., reduced T-lymphocyte CD4+ and increased natural killer cells), with an impaired response to specific antigens [57,60]. Direct mechanisms such as lymphocyte redox imbalance [26] and stress hormone production [49] might contribute to impaired lymphocyte function during IT recovery. However, lymphocytes might adapt to repeated IT sessions and become more resistant to stress [27,42]. Moreover, impaired lymphocyte function is transient and returns to basal levels a few hours after exercise cessation [26,27,57,60]. Apoptosis of high differentiated lymphocyte T-cells after an acute IT session concomitantly with increased Treg cell count and progenitor cells suggest that IT could acutely impair immune response against latent infection, while improving immune defense against new invading infectious agents [49].

IT performed in both alternate [27,42] and consecutive days [67] might improve lymphocyte function. In contrast, IT performed until exhaustion in consecutive days may impair immune restoration and exacerbate lymphocyte migration and apoptosis [29]. These controversial results might be explained by diversity of IT protocols [7], which might result in different physiological responses [73,74,75]. Of note, studies reporting chronic improvements in immune system have reported no changes or reduction in the levels of stress hormones (e.g., cortisol and catecholamines) over long term [42,67]. This suggests that there was appropriate interval rest between IT sessions, since insufficient recovery is associated with chronic increases of these hormone levels at rest [76]. The adequate recovery might be dependent of IT type, protocols that promote higher increases in oxidative stress and stress hormone response may require more time to immune system restoration in comparison with less stressful IT protocols.

In short term, IT contribute to increase the Treg frequency in individuals with impaired metabolic profile (e.g., men with obesity) [41]. These findings are particularly important since Treg plays a key role on immune function regulation and its low levels are associated with impaired immune response [77]. Although IT may acutely have a negative impact on immune functioning, the increased susceptibility for illness seems be more associated with training schedule than an IT session per se. Considering its physiological demand, high training frequency or insufficient recovery between IT session might contribute to increased illness risk [29], while proper IT prescription might provide increased physical performance concomitantly with improvements or preservation of immune system [28,78].

As a practical recommendation, IT protocols that promotes elevated metabolic stress (e.g., high levels of cortisol, lactate, and sympathetic activation) should be avoided when it is desirable to preserve immune function such as in patients with depressed immune function or at imminent infection risk. In this sense, IT protocols involving short bouts (≤60 s) at submaximal efforts (≤90% of parameter associated with ($\dot{V}$O_2max_) and total sessions with duration no longer than 60 min seems to be recommended. Whereas IT sessions involving “all out” efforts (≥30 s) seem to promote a greater disturbance on immune system.

There is compelling evidence that cardiorespiratory fitness is closely associated or can modulate immune functioning [28,41,79,80,81,82,83], such that improved cardiorespiratory fitness might decrease the risk of illness. Therefore, a proper IT prescription might provide a time-efficient strategy to increase cardiorespiratory fitness while preserve or improve immunological function. In this sense, the detailed description of the effects of different IT types on several immunological parameters might contribute to provide valuable findings regarding proper IT prescription in immune system context. Whereas some acute parameters change after IT seem not clinically relevant per se, to understand their behavior should contribute to the maintenance of a sustainable exercise routine over the medium and long term.

This systematic review with meta-analysis was not free from limitations. Inclusion criteria resulted in heterogeneous studies, and conclusions could not be made to a specific effect of IT on immune system. In this sense, the variety of IT protocols, study designs, and outcomes might compromise the external validity of our analysis. Secondly, most studies included in this systematic review with meta-analysis have low quality and used relatively small sample sizes. Future studies should improve their methodological quality to provide reliable conclusions regarding the effects of IT on immunity. While further research is warranted to investigate the association between IT and illness risk. On the other hand, to the author’s knowledge, this is the first study to summarize the state-of-the-art knowledge available currently, regarding the effect of IT on immune system, which might bring relevant contributions to research area and clinical practice.

## 5. Conclusions

Based on our systematic review with meta-analysis of available literature, a single session of IT might provide a transient disturbance on the immune system, followed by reduced immune function. On the other hand, regular IT performance induces favorable adaptations on immune function, improving immunosurveillance in the short to long term without changing immune cell count.

## Figures and Tables

**Table 1:** Summarize of studies investigating the acute effects of interval training on immune outcomes.

Study	Participants	Design	Modality/Interval Protocol	Results
Monje et al. 2020 [43]	20 runners (10 men age: 21.9 ± 0.8 years; 10 women age: 25.8 ± 6.2 years)	Clinical trial	Running HIIT—10 bouts of 4 min at 90% of v$\dot{V}$O_2max_ interspersed by 2 min of passive recovery	↑ salivary IgA concentration 20 min after exercise
Wahl et al. 2020 [44] 12 men triathletes and cyclists (age: 24.7 ± 3.4 years) Randomized cross-over trial			Cycling HIIT—4 bouts of 4 min at 90–95% of peak power interspersed by 3 min of passive recovery	↔ leucocyte count; ↓ lymphocyte count 30 min, and 60 min after exercise; ↑ neutrophil count 180 min after exercise; ↔ mixed cell count
			Cycling HIIT—4 bouts of 4 min at 90–95% of peak power interspersed by 3 min at 45% of peak power	↔ leucocyte count; ↑ lymphocyte count immediately after exercise followed by ↓ 30 min, 60 min and 180 min after exercise; ↑ neutrophil count 60 min and 180 min after exercise; ↔ mixed cell count
			Cycling SIT—4 bouts of 30 s “all-out” effort interspersed by 7.5 min passive recovery	↑ leucocyte count immediately, and 180 min after exercise; ↑ lymphocyte count immediately after exercise followed by ↓ 60 min and 180 min after exercise; ↑ neutrophil count 60 min and 180 min after exercise; ↑ mixed cell count immediately after exercise
			Cycling SIT—4 bouts of 30 s “all-out” effort interspersed by 7.5 min at 45% of peak power	↑ leucocyte count immediately, and 180 min after exercise; ↑ lymphocyte count immediately after exercise followed by ↓ 30 min, 60 min, and 180 min after exercise; ↑ neutrophil count 60 min, and 180 min after exercise; ↔ mixed cell count
De Oliveira Ottone et al. 2019 [25]	12 inactive health men (age: 22.5 ± 3.9 years)	Clinical trial	Cycling HIIT—8 bouts of 60 s at 90% peak power interspersed by 75 s of active recovery (30 watts)	↓ neutrophil oxidative burst in response to f-PMN 30 min after exercise; ↑ neutrophil phagocytic capacity, oxidative burst and redox status 24 h after exercise
Jamurtas et al. 2018 [45]	12 health men (age: 22.4 ± 0.5 years)	Randomized cross-over trial	Cycling SIT—4 bouts of 30 s “all-out” effort interspersed by 4 min of active recovery	↑ leucocyte count immediately after exercise
Souza et al. 2018 [46]	10 obese men (age: 28.5 ± 2.7 years)	Randomized cross-over trial	Running HIIT—10 bouts of 1 min at 90% of V_max_ interspersed by 1 min at 30% of V_max_	↔ secretory IgA and IgA concentration
Rodrigues de Araujo et al. 2018 [47]	32 men soccer players (age: 21.2 ± 4.2 years)	Clinical trial	Running SIT—7 bouts of 40 m “all-out” effort with direction changes interspersed by 25 s of active recovery (light jogging)	↔ IgA concentration
Belviranli et al. 2017 [48]	10 inactive health men (age: 20.0 ± 1.33 years)	Clinical trial	Cycling SIT—4 bouts of 30 s “all-out” effort interspersed by 4 min of active recovery (the load was determined according with the Monark Anaerobic Test Software)	↑ leucocyte count immediately, 3h, and 6 h after exercise; ↑ lymphocyte count immediately after exercise followed by ↓ lymphocyte count 3 h, and 6 h after exercise; ↑ neutrophil count 3 h, and 6 h after exercise; ↔ monocyte count; ↑ eosinophil count immediately after exercise followed by ↓ 3 h, and 6 h after exercise; ↑ basophil count immediately after exercise
Krüger et al. 2016 [49]	23 untrained health men (age: 25.7 ± 3.2 years)	Randomized cross-over trial	Cycling HIIT—5 bouts of 3 min at 90% peak power output interspersed by 3 min of active recovery (without resistance)	↑ lymphocyte CD3+, CD4+ and CD8+ count immediately, and 3 h after exercise; ↑ mobilization of low differentiated T cells, regulatory T cells and progenitor cells; ↑ apoptosis in high differentiated T cells
Tossige-Gomes et al. 2016 [26]	10 inactive health men (age: 23.7 ± 1.1)	Clinical trial	Cycling HIIT—8 bouts of 1 min at 100% of peak power interspersed by 75 s of active recovery at 30 W	↑ lymphocyte redox imbalance 30 min after exercise; ↓ lymphocyte proliferation in response to antigenic, but not to mitogenic stimulation immediately and 30 min after exercise
	6 inactive health men (age: 21.3 ± 1.8 years)		Cycling HIIT—8 bouts of 1 min at 100% of peak power interspersed by 75 s of active recovery at 30 W	↔ lymphocyte viability
Turner et al. 2016 [50]	9 health men (age: 22.1 ± 3.4 years)	Randomized cross-over trial	Cycling HIIT—10 bouts of 1 min at 90% of $\dot{V}$O_2max_ interspersed by 1 min at 40% of $\dot{V}$O_2max_	↑ leucocyte, lymphocyte count immediately after exercise; mobilization of cutaneous lymphocyte natural killer and lymphocyte CD8+ to blood
Dorneles et al. 2016 [51]	12 overweight-obese men (age: 27.41 ± 9.20 years)	Randomized cross-over trial	Running HIIT—10 bouts of 1 min at 85–90% maximum power output interspersed by 75 s at 50% maximum power output	↑ leucocyte, lymphocyte, and monocyte count immediately after exercise
	10 lean men (age: 26.5 ± 6.11 years)		Running HIIT—10 bouts of 1 min at 85–90% maximum power output interspersed by 75 s at 50% maximum power output	↑ leucocyte immediately and 30 min after exercise; ↑lymphocyte and monocyte immediately after exercise
Arroyo-Morales et al. 2012 [52]	50 active health subjects, 25 men (age: 22.4 ± 3.42 years)	Clinical trial	Arm-cycling SIT—3 bouts of 30 s “all-out” effort interspersed by 3 min (90 s of active recovery at 50% W work rate and 90 s of passive recovery)	↔ secretory IgA
Friedman et al. 2012 [53]	8 health subjects, 4 men (age: 24)	Clinical trial	SIT—2 sets of 3 bouts of 30 s “all-out” effort interspersed by 2 min of active recovery. Sets were separated by 6.75 min	↑ lymphocyte CD8+, and CD8+/CD45RA+ count and ↑ lymphocyte CD8+, and CD8+/CD45RA+ migration immediately after exercise. ↑ lymphocyte CD8+, and CD8+/CD45RA+ count and ↔ lymphocyte CD4+, and CD4+/CD45RA+ migration immediately after exercise
Fisher et al. 2011 [27]	8 active health men (age: 22 ± 2 years)	Clinical trial	Cycling HIIT—4 bouts with 30 s at 90% of maximum anaerobic power interspersed by 4 min of active recovery at 15% of maximum anaerobic power	↑ leucocyte and neutrophil counts immediately and 3 h after exercise; ↑ lymphocyte count immediately after exercise; ↓ lymphocyte cell viability 3 h after exercise
Davison 2011 [24]	9 active health men (age: 27 ± 5 years)	Randomized cross-over trial	Cycling SIT—4 bouts of 30 s “all-out” effort interspersed by 4 min of active recovery with light loads	↔ secretory IgA and ↑ IgA concentration; ↑ neutrophil count immediately and 30 min after exercise; ↓ neutrophil oxidative burst in response to fMLP 30 min after exercise
Thomas et al. 2010 [54]	10 health adolescent women (age 15.5 ± 0.6 years)	Clinical trial	Cycling SIT—8 bouts of 8 s “all-out” effort interspersed by 30 s of passive recovery	↔ IgA concentration 5 min after exercise
Fahlman et al. 2001 [22]	26 active health women (age: 24.2 ± 5.8 years)	Clinical trial	Cycling SIT—3 bouts of 30 s “all out” effort interspersed by 3 min (90 s of active recovery pedaling against light load and 90 s of passive recovery)	↓ secretory IgA and ↔ IgA concentration 5 min after exercise
Walsh 1999 [55]	8 trained men (age: 25 ± 1 years)	Clinical trial	Cycling HIIT –20 bouts of 1 min at 100% of $\dot{V}$O_2max_ interspersed by 2 min at 30% of $\dot{V}$O_2max_	↔ secretory IgA and IgA concentration after exercise
Walsh et al. 1998 [56]	8 trained men (age: 25 ± 3 years)	Clinical trial	Cycling HIIT—20 bouts of 1 min at 100% of $\dot{V}$O_2max_ interspersed by 2 min at 30% of $\dot{V}$O_2max_	↑ leucocytes and neutrophil count 5 min, 1 h, 2.5 h, and 5 h after exercise; ↑ lymphocyte count immediately after exercise followed by ↓ 1 h after exercise
Hinton et al. 1997 [57]	5 men runners (age: 23.0 ± 2.5 years)	Clinical trial	Running HIIT—15 bouts of 1 min at 90% of $\dot{V}$O_2max_ interspersed by 2 min of passive recovery	↓ lymphocyte function immediately after exercise
Kargotich et al. 1997 [58]	8 high performance men swimmers (age: 19.9 ± 2.2 years)	Clinical trial	Swimming HIIT—15 bouts of 100 m freestyle swimming interspersed by 2 min 25 m recovery swim	↑ leucocyte and neutrophil count immediately after exercise; ↑ lymphocyte count immediately after exercise followed by ↓ 1 h, 2 h, and 2.5 h after exercise; ↑ monocyte count immediately and 30 min after exercise; ↔ eosinophil count
Gray et al. 1993 [59]	8 men triathletes (age: 31.5 ± 4.5 years)	Clinical trial	Running HIIT—1 min at 100% of v$\dot{V}$O_2max_ interspersed by 1 min of active recovery until the exhaustion	↑ leucocyte and lymphocyte count immediately after exercise; ↑ granulocyte and monocyte count 6 h after exercise
Mackinnon & Jerkin, 1993 [23]	12 active health men (age: 17 to 25 years)	Clinical trial	Cycling SIT—5 bouts of 1 min “all out” effort interspersed by 5 min of passive recovery	↓ secretory IgA and ↑ IgA concentration immediately after exercise
Fry et al. 1992 [60]	14 men runners (age: 18–25 years)	Clinical trial	Running Treadmill HIIT—25 bouts of 1 min at one stage before that which the subject failed in the preliminary test) followed by 2 min active recovery	↓ lymphocyte proliferative response immediately after exercise
	18 men kayakists (age: 18–25 years)		Paddling HIIT—25 bouts of 1 min at one stage before that which the subject failed in the preliminary test interspersed by 2 min of active recovery	↓ lymphocyte proliferative response immediately after exercise
Fry et al. 1992 [61]	7 men runners (age: 22.9 ± 5.6 years)	Cross-over clinical trial	Running HIIT—15 bouts of 1 min at 90% of V_max_ interspersed by 2 min of active recovery	↔ leucocytes, lymphocyte, neutrophil and monocyte count 5 min after exercise. ↔ the CD4^+^:CD8^+^ ratio and responsiveness of T cells to T cells mitogens
			Running HIIT—15 bouts of 1 min at 120% of V_max_ interspersed by 2 min of active recovery	↑ leucocytes count, lymphocyte, neutrophil, monocyte count 5 min after exercise. ↓ the CD4^+^:CD8^+^ ratio and responsiveness of T cells to mitogens immediately after exercise

HIIT, high intensity interval training; SIT, sprint interval training; IgA, immunoglobulin A; $\dot{V}$O_2max_, maximal oxygen consumption; $\dot{V}$O_2_max, velocity associated to maximal oxygen consumption; V_max_, maximal velocity achieved during the incremental test. fMLP, formyl-leucyl-methionyl-phenylalanine. ↑ significant increase; ↓ significant decrease; ↔ no significant change.

**Table 2:** Summarize of studies investigating the chronic effects of interval training on immune outcomes.

Study	Participants	Duration/Design	Modality/Interval Protocol	Results
Bartlett et al. 2020 [62]	10 subjects with prediabetes, 4 men (age: 71 ± 5 years)	Ten weeks clinical trial	Walking HIIT—60–90 s at 80–90% of $\dot{V}$O_2_ reserve interspersed by 60–90 s of active recovery at 50–60% of VO_2_ reserve until complete 20 min. Frequency: 3 times per week. Supervised: Yes	↑ neutrophil chemotaxis, mitogen stimulated ROS production and ↓ basal ROS production. ↔ neutrophil count
Toohey et al. 2020 [63]	6 breast cancer survivors (age: 60 ± 8.12 years)	Twelve weeks randomized clinical trial	Cycling SIT—4 to 7 bouts of 30 s “all-out” effort interspersed by 2 min of active recovery. Frequency: 3 times per week. Supervised: Yes	↔ IgA concentration
Dorneles et al. 2019 [41]	7 sedentary obese men (age: 20 to 40 years)	One-week clinical trial	Running HIIT—10 bouts of 1 min at 85–90% maximum heart rate interspersed by 75 s at 50% maximum heart rate. Frequency: 3 times per week. Supervised: No reported	↑ circulating of memory regulatory T cells and regulatory T cells
Werner et al. 2019 [42]	29 inactive health subjects, 10 men (age: 48.4 ± 6.5 years)	Twenty-six weeks randomized controlled trial	Running HIIT—4 bouts of 4 min at 80–90% of heart rate reserve interspersed by 3 min at 65–70% of heart rate reserve. Frequency: 3 times per week. Supervised: No reported	↔ total leucocyte counts (lymphocyte, neutrophil and monocyte); ↑ leucocyte telomerase length (lymphocyte, granulocyte)
Khammassi et al. 2020 [30]	8 active health young adults (age: 18.9 ± 1.0 years)	Nine weeks randomized clinical trial	Running HIIT—3 sets of 6 to 8 30-s bouts at 100 to 110% of V_max_ and 30 s of active recovery at 50% of V_max._ Frequency: 3 times per week. Supervised: No reported	↔ total leucocyte counts (lymphocyte, neutrophil and monocyte)
Bartlett et al. 2018 [64]	12 inactive elderly subjects with rheumatoid arthritis (age: 64 ± 7 years)	Ten weeks clinical trial	Walking HIIT—60–90 s at 80–90% of $\dot{V}$O_2_ reserve interspersed by active recovery with similar duration at 50–60% of VO_2_ reserve until complete 20 min of session. Frequency: 3 times per week. Supervised: Yes	↑ neutrophil function
Sheykhlouvand et al. 2018 [65]	7 men canoe polo athletes (age: 24 ± 3 years)	Three weeks randomized clinical trial	Paddling HIIT—6 bouts of 1 min at 100 to 130% v$\dot{V}$O_2peak_ with 1:3 work to recovery ratio. Frequency: 3 times per week. Supervised: No reported	↔ leucocyte counts
	7 men canoe polo athletes (age: 24 ± 3 years)		Paddling HIIT—6 to 9 bouts of 1 min at 100% v$\dot{V}$O_2_peak with 1:3 work to recovery ratio. Frequency: 3 times per week. Supervised: No reported	↔ leucocyte counts
Bartlett et al. 2017 [66]	14 inactive health adults (age: 43 ± 11 years)	Ten weeks randomized clinical trial	Cycling HIIT—15 to 60 s above 90% of maximum heart rate interspersed by 45–120 s of active recovery until complete 18–25 min. Frequency: 3 times per week. Supervised: Yes	↑ neutrophil and monocyte function
Tsai et al. 2016 [67]	20 inactive health men (age: 23.0 ± 1.7 years)	Six weeks randomized clinical trial	Cycling HIIT—5 bouts of 3 min at 80% of $\dot{V}$O_2max_ interspersed by 3 min of active recovery at 40% of $\dot{V}$O_2max._ Frequency: 5 times per week. Supervised: No reported	↑ lymphocyte function
Navalta et al. 2014 [29]	12 subjects, 8 men (age: 26 ± 4 years)	Three consecutive days clinical trial	Running HIIT—30 s at 100% of Vmax interspersed by active recovery with similar duration at 50% of V_max_ until exhaustion. Frequency: 3 times per week. Supervised: No reported	↑ lymphocyte apoptosis
Fisher et al. 2011 [27]	8 active health men (age: 22 ± 2 years)	One-week clinical trial	Cycling HIIT—4 bouts with 30 s at 90% of maximum anaerobic power interspersed by 4 min of active recovery at 15% of maximum anaerobic power. Frequency: 3 times per week. Supervised: No reported	↑ lymphocyte function
Mackinnon & Jerkin, 1993 [23]	12 active health men (age: 17 to 25 years)	Eight weeks clinical trial	Cycling SIT—5 bouts of 1 min “all out” effort interspersed by 5 min of passive recovery. Frequency: 3 times per week. Supervised: Yes	↔ secretory IgA and IgA concentration

HIIT, high intensity interval training; SIT, sprint interval training; IgA, immunoglobulin A; $\dot{V}$O_2max_, maximal oxygen consumption; v$\dot{V}$O_2_max, velocity associated to maximal oxygen consumption; V_max_, maximal velocity achieved during the incremental test. ROS, reactive oxygen species; ↑ significant increase; ↓ significant decrease; ↔ no significant change.

**Table 3:** Study quality and reporting of randomized clinical trial included studies.

Reference	Study Quality					Score (0–5)	Study Reporting										Score (0–10)	Total Score (0–15)	Study Quality Classification
	1	2	3	4	5		6a	6b	6c	7	8a	8b	9	10	11	12			
Khammassi et al. [30]	+	−	+	+	−	3	−	−	−	−	−	−	+	NA	−	+	2	5	Low
Toohey et al. [63]	+	+	+	−	+	4	+	−	+	−	+	+	+	−	+	+	7	11	High
Wahl et al. [44]	−	−	−	−	−	0	−	−	−	−	+	+	+	NA	−	+	4	4	Low
Dorneles et al. [41]	+	−	−	−	−	1	−	−	−	−	−	−	+	NA	+	+	3	4	Low
Werner et al. [42]	+	−	+	+	−	3	−	−	−	−	+	+	+	−	−	+	4	7	Low
de Souza et al. [46]	+	+	−	−	−	2	−	−	−	−	+	+	+	−	−	+	4	6	Low
Jamurtas et al. [45]	−	−	−	−	−	0	−	−	−	−	−	−	+	NA	−	+	2	2	Low
Sheykhlouvand et al. [65]	+	−	+	−	−	2	−	−	−	−	−	−	+	NA	−	+	2	4	Low
Bartlett et al. [66]	−	−	+	+	−	2	−	−	−	−	−	−	+	NA	−	+	2	4	Low
Krüger et al. [49]	+	−	−	−	−	1	−	−	−	−	−	−	−	NA	−	+	1	2	Low
Tsai et al. [67]	+	−	−	−	−	1	+	−	+	−	+	+	+	+	+	+	8	9	Fair
Turner et al. [50]	−	−	−	−	−	0	−	−	+	−	−	−	+	NA	−	+	3	3	Low
Davison. [24]	−	−	−	−	−	0	−	−	−	−	+	+	+	−	−	−	3	3	Low

+, meet the criteria; −, do not meet the criteria; NA, not applicable.

**Table 4:** Subgroup analysis of overall effects of interval training on immune outcomes.

Outcome (Subgroup)	N° of Studies	MD (95% CI)	*p*-Value	Heterogeneity	
				I^2^ (%)	*p*-Value
IgA concentration (µg·mL^−1^)					
IT type: SIT	5	46.98 (56.73 to 150.68)	0.37	94	<0.001
IT type: HIIT	**4**	**39.54 (19.92 to 59.16)**	**<0.001**	**0**	**1**
Sex: men	6	65.62 (−6.43 to 137.66)	0.07	91	<0.001
Sex: women	3	−18.91 (−66.24 to 28.42)	0.43	0	0.59
Modality: cycling	5	53.22 (−33.53 to 139.96)	0.23	94	<0.001
Modality: running	4	22.07 (−17.34 to 61.47)	0.27	0	0.92
IgA secretory rate (µg·min^−1^)					
IT type: SIT	**6**	**−17.33 (−33.68 to −0.98)**	**0.03**	**68**	**0.007**
IT type: HIIT	2	−7.29 (−23.95 to 9.36)	0.39	0	0.73
Sex: men	5	−13.17 (−35.03 to 8.70)	0.24	74	0.004
Sex: women	**2**	**−19.34 (−29.11 to −9.58)**	**<0.001**	**0**	**0.74**
Modality: cycling	-	-	-	-	-
Modality: running	-	-	-	-	-
Leucocyte count (10^3^ µL^−1^)					
IT type: SIT	**5**	**3.14 (1.83 to 4.44)**	**<0.01**	**80**	**<0.001**
IT type: HIIT	**9**	**2.31 (1.30 to 3.32)**	**<0.001**	**78**	**<0.001**
Sex: men	-	-	-	-	-
Sex: women	-	-	-	-	-
Modality: cycling	**9**	**2.40 (1.47 to 3.33)**	**<0.001**	**84**	**<0.001**
Modality: running	**3**	**2.46 (1.30 to 3.62)**	**<0.001**	**21**	**0.28**
Lymphocyte count (10^3^ µL^−1^)					
IT type: SIT	**3**	**1.62 (0.89 to 2.35)**	**<0.001**	**66**	**0.05**
IT type: HIIT	**9**	**1.21 (0.67 to 1.74)**	**<0.001**	**81**	**<0.001**
Sex: men	-	-	-	-	-
Sex: women		-	-	-	-
Modality: cycling	**8**	**1.17 (0.65 to 1.70)**	**<0.001**	**82**	**<0.001**
Modality: running	**3**	**1.14 (0.67 to 1.61)**	**<0.001**	**10**	**0.33**
Lymphocyte count (10^3^ µL^−1^) recovery					
IT type: SIT	**3**	**−0.51 (−0.77 to −0.26)**	**<0.001**	**18**	**0.30**
IT type: HIIT	9	−0.29 (−0.56 to 0.03)	0.03	66	0.003
Sex: men	-	-	-	-	-
Sex: women	-	-	-	-	-
Modality: cycling	**8**	**−0.47 (−0.62 to −0.32)**	**<0.001**	**0**	**0.55**
Modality: running	3	0.04 (−0.63, 0.72)	0.9	85	0.001

SIT, sprint interval training; HIIT, high-intensity interval training. Significant *p*-values are indicated in bold.

## Data Availability

Not applicable.
